# Large language models for neurology: a mini review

**DOI:** 10.3389/fdgth.2025.1732759

**Published:** 2026-01-06

**Authors:** Donald C. Wunsch III, Daniel B. Hier

**Affiliations:** 1 Saint Louis University School of Medicine, St. Louis, MO, United States; 2 Center for Artificial Intelligence and Autonomous Systems, Kummer Institute, Missouri University of Science and Technology, Rolla, MO, United States

**Keywords:** ambient documentation, digital twins, documentation burden, ethical AI, large language models, multimodal AI, neurology, precision neurology

## Abstract

Large language models have the potential to transform neurology by augmenting diagnostic reasoning, streamlining documentation, and improving workflow efficiency. This Mini Review surveys emerging applications of large language models in Alzheimer’s disease, Parkinson’s disease, multiple sclerosis, and epilepsy, with emphasis on ambient documentation, multimodal data integration, and clinical decision support. Key barriers to adoption include bias, privacy, reliability, and regulatory alignment. Looking ahead, neurology-focused language models may develop greater fluency in biomedical ontologies and FHIR standards, improving data interoperability and supporting more seamless collaboration between clinicians and AI systems. Two future developments have the potential to be particularly impactful: (1) the integration of multi-omic and neuroimaging data with digital-twin simulations to advance precision neurology, and (2) broader adoption of ambient documentation and other language-model–based efficiencies that could reduce administrative and cognitive burden. Ultimately, the clinical success of large language models will depend on continued progress in model robustness, ethical governance, and careful implementation.

## 1 Introduction

The ascent of large language models marks a tipping point in clinical medicine, integrating clinical informatics, artificial intelligence (AI), data analytics, and precision medicine into a unified paradigm [1]. These models now leverage deep learning architectures to process vast datasets, interpret complex clinical relationships, and generate human-like text [2]. As their scale and contextual depth have expanded, large language models—originally designed as next-token predictors—have acquired emergent capabilities [3]: functioning as repositories of medical knowledge, summarizers of clinical text, and reasoning engines that exhibit physician-like competence in diagnosis, prognosis, and therapeutics. Neurology, characterized by complex diagnostic reasoning, detailed clinico-anatomic correlation, extensive unstructured documentation, and integration of multimodal data streams including radiologic and electrophysiologic inputs, is uniquely positioned to benefit from their entry into clinical medicine [4].

The American Academy of Neurology (AAN) has released a position statement on the use of large language models in neurology [1]. Their stance is hopeful yet disciplined: these models are framed as a potential breakthrough in neurological efficiency and quality—provided they are adopted deliberately, validated rigorously, and governed ethically. The emphasis is on *responsible innovation*, embracing the transformative potential of the technology while acknowledging its immaturity and risks in real-world practice. The AAN statement expresses enthusiasm for the promise of large language models to improve documentation, reduce administrative burden, and enable quality measurement, yet tempers this optimism with concerns about safety, bias, reliability, and governance. Avoiding both technological pessimism and uncritical endorsement, the statement characterizes these systems as powerful but immature tools requiring robust oversight, transparent validation, and ethical governance.

We performed a targeted literature search using Google Scholar (https://scholar.google.com/) and Consensus GPT (https://consensus.app/) to identify key primary publications on large language models in neurology, using the search terms large language models AND neurology. We also conducted forward citation tracking to identify subsequent articles citing these foundational studies, resulting in a core list of 31 relevant publications (Table 1). The primary search covered papers published from January 2023 through November 2025. Papers were classified by topic area (e.g., Diagnosis, Documentation, Management) and by type (Review, Research, Opinion). Additional articles were included as needed to ensure completeness of the Mini Review. Papers were selected based on relevance to neurology, contribution to understanding LLM capabilities, and overall quality as independently judged by both co-authors. This process yielded a focused—though not exhaustive—survey appropriate for a Mini Review.

**Table 1:** Core references utilized in mini review.

Author	Year	Topic	Type	Comment
Jones	2022	AI in neurology	Opinion	Forecast for 2035
Moura	2024	AI in neurology	Opinion	AAN position statement
Rizzo	2025	AI in neurology	Review	Comprehensive overview
Romano	2023	AI in neurology	Opinion	Ethical challenges
Westover	2025	AI in neurology	Opinion	Risks vs. benefits
Barrit	2025	Diagnosis	Research	LLM outperforms neurologists
Cano-Besquet	2024	Diagnosis	Research	LLMs comparable to neurologists
Ford	2024	Diagnosis	Research	Limited accuracy on seizures
Habibi	2025	Diagnosis	Research	Limited accuracy
Joseph	2024	Diagnosis	Research	Limited accuracy for MS
Maiorana	2025	Diagnosis	Research	Neurologists outperform LLMs
Qadri	2024	Diagnosis	Review	Dementia diagnosis
Song	2025	Diagnosis	Research	Stroke diagnosis from notes
Sorka	2025	Diagnosis	Research	LLM outperformed neurologists
Twala	2025	Diagnosis	Research	Multimodal ML for parkinsonism
Yang	2024	Diagnosis	Research	LLM identifies seizure locus
Zamai	2025	Diagnosis	Research	Dementia MRI interpretation
Chadehumbe	2025	Documentation	Research	Workflow with ambient AI
Ge	2023	Documentation	Opinion	Benefits vs. risks
Chiang	2024	Localization	Opinion	Cautious optimism
Dani	2025	Localization	Research	Localizes seizure zones
Lee	2024	Localization	Research	High accuracy on stroke cases
Amin	2024	Disease management	Review	MS management
Harrison	2025	Disease management	Review	Dementia management
Naji	2023	Disease management	Review	MS management
Mavrych	2025	Question answering	Research	Advanced LLMs did better
Ros-Arlanzon	2024	Question answering	Research	GPT-4 outperformed GPT-3.5
Schubert	2023	Question answering	Research	LLMs outperformed humans
Shojaee-Mend	2024	Question answering	Research	Limitations noted
Shu	2024	Question answering	Research	LLM outperformed humans

LLM, Large Language Model; MS, Multiple Sclerosis; MRI, Magnetic Resonance Imaging; ML, Machine Learning; AAN, American Academy of Neurology.

All works listed in this table are cited in the main text and appear in the reference list.

In this Mini Review, we examine how large language models may improve neurological care and reduce documentation and administrative burdens. We review assessments of neurological knowledge, including foundational neuroscience understanding, lesion localization, and diagnostic reasoning. We then discuss their role in workflow efficiency, disease management, and the challenges that accompany their implementation. Finally, we present our predictions for the state of neurology-focused large language models in 2035 [5].

## 2 Assessing neurological competency in large language models

Large language models have demonstrated emerging capabilities across several domains of neurology—including board—style question answering, neurological diagnosis, and lesion localization. Proficiency in these domains is essential for establishing the credibility and clinical utility of a large language model.

### 2.1 Neurological knowledge

Large language models have demonstrated substantial progress in mastering the foundational knowledge base of neurology, as reflected in their performance on standardized board-style examinations. GPT-4 correctly answered 82% of U.S. neurology board-style questions (*n* = 1,956), surpassing GPT-3.5 (66%) and approaching the lower range of specialist performance [6]. Three large language models (Bard, Claude 2, and GPT-3.5) were evaluated on 20 essay-style advanced neurophysiology questions, which were scored by physiologists on a 0–5 scale, yielding a mean score of 3.9/5 across models [7]. On the Spanish Neurology Specialist Examination (77 multiple-choice questions), GPT-4 scored 81.8% correct—ranking seventeenth among 120 neurologists who took the examination [8]. On 200 multiple-choice questions comparable to the neuroscience section of the United States Medical Licensing Examination (USMLE), Claude (88.0%) and GPT-4 (81.7%) outperformed the student average (74.6%) [9]. On the NeuroReady® board preparation question bank (*n* = 400), GPT-4 scored 75%, exceeding the passing threshold (70%) and the average test-taker score (69%) [10]. Collectively, these findings suggest that advanced large language models such as GPT-4 have reached near-human competence in factual neurology knowledge [6–12].

### 2.2 Neurological lesion localization

Accurate lesion localization based on signs and symptoms remains a defining cognitive skill of neurologists [13–15]. In a structured evaluation of 46 acute stroke vignettes derived from published cases, GPT-4 localized brain lesions with an F1 score of 0.85 for brain region and 0.74 for lesion side. Although no direct human comparison was performed, performance varied by region, with best results for cerebral and spinal lesions and poorest for cerebellar lesions [16]. Most localization errors arose from incomplete input data or reasoning gaps rather than factual hallucination, supporting GPT-4’s potential for structured neuroanatomical reasoning.

### 2.3 Neurological diagnosis

The diagnostic accuracy of large language models in neurology varies by setting and model. Current evidence suggests that while general-purpose large language models lag behind human neurologists in real-world practice, domain-specialized models can approach or exceed human performance in selected scenarios.

In a comparative study using 28 real-world anonymized patients, neurologists achieved substantially higher diagnostic accuracy (75%) than general-purpose large language models such as GPT-3.5 (54%) and Gemini (46%) [17]. These models struggled with complex clinical reasoning, contextual integration, and subtle diagnostic differentiation.

Similarly, OpenBioLLM—a domain-specialized model—achieved only 38% diagnostic accuracy on 25 cases from the textbook *Clinical Cases in Neurology*. Although the model frequently identified relevant symptoms and correctly localized the lesion, it often failed to arrive at an accurate etiologic diagnosis [18].

In contrast, the neurology-specialized model *Neura* demonstrated substantially higher diagnostic capability [19]. In a blinded comparison with 13 neurologists using five difficult *Clinical Reasoning* cases from the journal **Neurology**, *Neura* achieved scores of 86% overall, 85% for differential diagnosis, and 88% for final diagnosis. Neurologists scored 55%, 46%, and 71%, respectively. *Neura* produced rapid, citation-backed responses with minimal hallucination.

General-purpose large language models have also shown strong performance in specific tasks such as seizure localization. When presented with 1,269 clinical epilepsy narratives, Mistral-8x7B (F1 = 51.7) and GPT-4 (F1 = 52.3) localized epileptogenic zones across seven brain regions as accurately as expert neurologists (F1 = 48.8) [20].

## 3 Neurologist workflow and efficiency

Large language models have the potential to improve the workflow and efficiency of neurologists. They can generate patient-specific educational materials in real-time by drawing directly from the electronic health record (EHR), reducing the need for manual customization [21]. In the outpatient setting, ambient AI systems can capture and document the patient encounter—recording the history, examination findings, assessment, and plan—thereby reducing documentation burden on the neurologist [22, 23]. In a pilot study, 10 of 13 neurology providers reported improved efficiency by implementing ambient AI for documentation of outpatient neurology visits [23]. Large language model—based summarization tools can expedite pre-visit preparation by synthesizing entire EHRs into concise, clinically relevant overviews. Additional efficiency gains are possible through faster creation of discharge summaries, accelerated drug authorizations, improved prior approval workflows, streamlined scheduling for diagnostic testing, and automated chart coding [24]. Together, these capabilities of large language models could meaningfully ease the administrative and documentation burden faced by neurologists.

## 4 Disease-specific applications

### 4.1 Stroke

Acute stroke management is time-critical and data-intensive, making it an ideal domain for integration with large language models. Modern stroke care produces large volumes of unstructured text—from triage notes, radiology findings, and procedural reports—that must be interpreted rapidly for treatment decisions [25]. Large language models optimized for stroke care can integrate clinical narratives with non-contrast CT findings to diagnose stroke, assess eligibility for intravenous thrombolysis, and detect large vessel occlusions. Song et al. [26] fine-tuned ChatGLM-6B based on 1,885 patients with and without stroke. Patients were divided into training and validation sets. The model distinguished between hemorrhage and infarction with 100% accuracy, identified large vessel occlusions with 80% accuracy, and screened patients for intravenous thrombolysis with 89.4% accuracy. The model input was a non-contrast CT scan and the clinical notes [26]. These findings highlight the potential of large language models to streamline time-sensitive stroke workflows, speed diagnosis, and enhance decision support.

### 4.2 Alzheimer’s disease and dementia

Large language models are increasingly applied to the diagnosis and management of neurodegenerative disorders, particularly Alzheimer’s disease. They show promise for early detection by identifying subtle, complex symptom patterns within unstructured clinical text that may escape human recognition. Harrison et al. [27] provide a comprehensive review of emerging applications of large language models in improving Alzheimer’s disease diagnosis. Qadri et al. [28] review the utility of large language models in the diagnosis of neurodegenerative disorders such as Alzheimer’s disease and Parkinson’s disease. In an innovative approach, Zamai et al. [29] used fine-tuned large language models to classify 615 MRI images into four diagnostic categories (normal, Alzheimer’s disease, frontotemporal dementia, and primary progressive aphasia). The authors first converted each MRI into a synthetic radiology-style text report (an image-to-text approach) and then fine-tuned a language model to interpret these reports. With fine-tuning, Qwen-3.1-8B achieved a balanced accuracy of 68.4%, outperforming GPT-4o (balanced accuracy 55.5%) on the same classification task [29].

### 4.3 Parkinson’s disease

The diagnosis of Parkinson’s disease relies heavily on clinical observation of motor symptoms, which can lead to delayed or uncertain diagnosis in early stages. Advanced AI frameworks that integrate deep learning with natural language processing have been developed to analyze voice, gait, and motor patterns, enabling detection of subtle features of parkinsonism that may escape routine clinical examination. Twala [30] evaluated a multimodal model that combined gait analysis, voice analysis, and visual motor assessments to classify 847 synthetic patient profiles as having Parkinson’s disease or not. Using this synthetic dataset, the system achieved a diagnostic accuracy of 94.2%. Although these results are preliminary and limited by reliance on synthetic data, they highlight the potential of multimodal AI systems to enhance early detection of Parkinson’s disease.

### 4.4 Multiple sclerosis

Large language models such as GPT-4 are being evaluated for classifying multiple sclerosis (MS) status based on clinical notes. When aligned with diagnostic frameworks such as the 2017 McDonald criteria, they achieve classification accuracies of up to 74% [31]. Venkatesh et al. [31] used GPT-4 to reclassify 125 patients (105 with MS, 10 with related disorders, and 10 healthy controls) based on their clinical notes that included laboratory findings. GPT-4 correctly classified 74% (93/125) patients, including 70% of patients with MS, 100% of related disorders, and 90% of healthy controls. Large language model–based systems are increasingly applied to prognosis, integrating MRI data and large clinical registries to identify key predictors of disease progression and treatment response in multiple sclerosis [32].

### 4.5 Epilepsy and seizures

In epilepsy, large language models have been evaluated for challenging diagnostic distinctions, such as differentiating epileptic seizures from functional or dissociative seizures. In a study using patient-generated symptom descriptions, Ford et al. [33] tested GPT-4 on 41 cases (16 epilepsy, 25 functional/dissociative seizures). In the zero-shot condition, GPT-4 achieved a balanced accuracy of 57% (κ=0.15), which improved to 64% (κ=0.27) after a single example (one-shot prompting). Additional examples (two- and three-shot) did not further improve performance. In contrast, three experienced neurologists achieved a mean balanced accuracy of 71% (κ=0.42). Notably, in the subset of 18 cases correctly diagnosed by all three neurologists, GPT-4 achieved a balanced accuracy of 81% (κ=0.66), suggesting that performance improves substantially when clinical descriptions are clear and internally consistent. Large language models have also been explored for presurgical planning. Fine-tuned systems can localize seizure origins to epileptogenic zones using clinical narratives, streamlining early stages of the preoperative workflow [34]. In addition, language models are increasingly used to assist with generating structured reports for electroencephalograms (EEG), electromyograms (EMG), and nerve conduction studies [25].

Taken together, these disease-specific applications should be viewed as exploratory rather than definitive. Most studies used small or moderate sample sizes, were conducted at single centers, or relied on synthetic data, vignettes, or retrospectively assembled datasets. External validation was limited, and few evaluations tested performance in real-time clinical workflows. As a result, the reported accuracies are best interpreted as suggestive of what large language models may be able to do under controlled conditions, rather than as evidence that they are ready for routine clinical deployment.

## 5 Challenges and controversies

The widespread adoption of large language models in neurology faces significant challenges and controversies that require debate and resolution.

### 5.1 Bias

Bias arises from imbalanced training data. The overrepresentation of specific populations, institutions, or languages produce systematic inequities in diagnosis, classification, and treatment recommendations [25, 35–37].

### 5.2 Privacy

Large language models risk re-identifying protected health information through memorization or inadvertent data exposure. Breaches, leaks, and technical vulnerabilities must be managed through robust encryption, audit trails, and data-minimization protocols [38].

### 5.3 Trust

Trust depends on reliability, validity, and explainability. Reliability ensures consistent results; validity aligns with clinical ground truth; and explainability enables oversight and clinician confidence [39–43].

### 5.4 Accurate model inputs

Neurological reasoning depends heavily on subtle bedside findings that must be correctly observed and documented by the clinician [13, 44]. Large language models can only reason over what is recorded. This reflects the classical Garbage In, Garbage Out (GIGO) principle: incomplete or imprecise clinical inputs propagate into incomplete or erroneous model outputs. No current AI system can substitute for the neurologist’s direct examination; the fidelity of LLM- or LMM-generated reasoning is ultimately constrained by the quality of the clinician’s initial observations.

### 5.5 Regulation

Some large language models may qualify as medical devices, requiring validation, traceability, and postmarket surveillance. Current regulatory frameworks remain incomplete, demanding coordinated oversight among developers, clinicians, and regulators [45–47].

### 5.6 Multimodal integration

Neurological diagnosis requires synthesizing text, imaging, and physiologic signals. While contemporary large language models (LLMs) are primarily trained on text and therefore depend on narrative descriptions of MRI, EEG, and EMG findings, emerging *large multimodal models* (LMMs) can natively integrate information from multiple data streams [48]. These multimodal architectures show promise in harmonizing radiologic, electrophysiologic, and textual inputs, potentially reducing the need for intermediate text-based summaries in the future [49–53]. Although the terminology is still evolving, and the boundary between LLMs and LMMs is not yet fixed, neurology is likely to benefit disproportionately from models that can directly process high-dimensional clinical signals.

### 5.7 Keeping neurology large language models current

Retraining and manual updates are slow and prone to catastrophic forgetting. Retrieval-augmented generation (RAG) architectures provide a more scalable solution, coupling dynamic knowledge sources with stable reasoning engines [54, 55].

### 5.8 Specialized vs. foundation models

Foundation models offer scalability and multimodal reasoning, but domain-specific models achieve higher accuracy and lower hallucination rates for clinical tasks [19].

### 5.9 From textbook knowledge to real-world usability

Large language models perform well on structured examinations, yet show variable accuracy in real clinical contexts. Real-world validation remains the decisive test of their readiness for clinical use [56].

### 5.10 Evolving skills that support neurologist workflow

Neurology large language models should target reduction of documentation burden, automation of text summarization, and seamless integration with the EHR. EHR burden remains one of the greatest challenges facing neurologists [57, 58]. The design of large language models for neurologists must reflect clinician priorities—not replace—neurologist expertise [59, 60].

## 6 Neurology large language models in 2035

**Figure 1:**
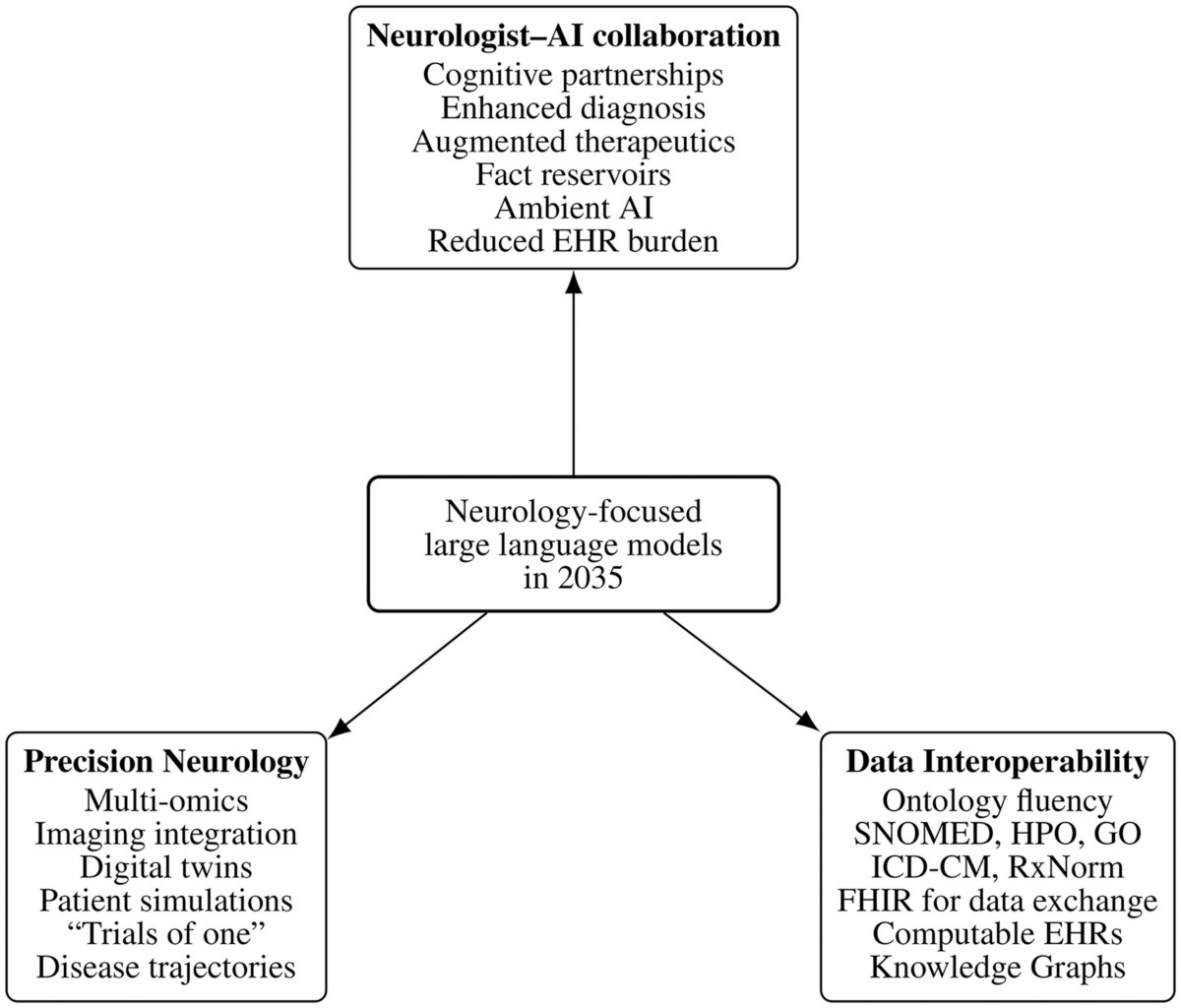
Conceptual overview of how neurology-focused large language models may transform neurology by 2035 along three major dimensions: (1) neurologist–AI collaboration, (2) precision and personalization through digital twins and multi-omic integration, and (3) ontology awareness and FHIR-native interoperability enabling a computable EHR.

We agree with Jones and Kerber [5] that, by 2035, neurology-focused large language models may evolve from static repositories of neurologic knowledge into more dynamic, ontology-aware computational tools that support neurological reasoning and synchronize with emerging biomedical information. Several developments could unfold along three major dimensions (Figure 1).

### 6.1 The evolving neurologist–AI collaboration

Large language models may function as active cognitive partners—suggesting differential diagnoses, proposing tests, or challenging initial hypotheses—while neurologists retain executive authority. The clinician’s role could shift subtly from synthesizing raw data toward validating and contextualizing AI recommendations, particularly in ambiguous or ethically complex cases [5, 25]. It is possible that advances in language-model–driven documentation will reduce the long-standing burdens associated with electronic health records, although the extent of such improvement will depend on technical reliability and clinical integration.

### 6.2 The emergence of precision and personalized neurology

Integration of digital twins and multi-omic data may enable more personalized disease trajectory forecasting and treatment selection [61–64]. Future multimodal frameworks could cross-validate predictions to reduce bias and achieve greater fluency across text, imaging, and physiological signal data [8]. AI-generated digital twins [65–67] might one day support *in silico* comparisons of therapeutic strategies—analogous to virtual “clinical trials of one”—for disorders such as multiple sclerosis or Parkinson’s disease. Whether such simulations will become routine clinical tools remains uncertain and will depend on regulatory oversight, validation, and clinician acceptance.

### 6.3 Infrastructure and interoperability for neurological data

A persistent barrier to real-world deployment of neurology-focused AI is the transformation of free-text clinical documentation into structured, computable, and interoperable data. Neurology-specific models may eventually achieve deeper fluency with biomedical ontologies—including SNOMED CT, HPO, GO, RxNorm, ICD-CM and with FHIR resource standards, thereby improving the consistency and computability of clinical data. Continuous alignment with curated databases and knowledge graphs may further reduce the need for frequent retraining, although such capabilities remain aspirational at present [68, 69]. Real progress will require: (1) consistent use of standardized vocabularies to encode diagnoses, medications, laboratory values, and neurological findings; (2) broad adoption of FHIR resources and profiles to represent encounters, observations, imaging studies, and procedures; and (3) robust NLP and mapping pipelines that convert narrative notes into coded concepts without losing clinically relevant nuance. Our preliminary work suggests that large language models can preprocess physician-written notes to improve downstream extraction of standard ontology terms and facilitate FHIR resource generation [70, 71]. Given that 60%–80% of clinically relevant information in U.S. electronic health records remains buried in free text [72, 73], expanding computability could meaningfully enhance both clinical care and research. However, this transition will require secure data platforms, governance frameworks, and auditable interfaces that allow AI systems to query and write back to the EHR safely. Without such infrastructure, even highly capable neurology models will remain confined to pilot settings rather than routine clinical practice.

## 7 Discussion

Before transformer-based large language models [74], artificial intelligence in neurology remained largely confined to narrow research prototypes. With the advent of large language models, diagnostic reasoning, documentation, and workflow assistance have moved from aspiration to implementation—a transition from theory to practice. Neurology has always been intellectually demanding, yet neurologists rarely cite cognitive challenge as their source of fatigue. Rather, burnout stems from administrative overload and documentation burden [57, 58]. Large language models now offer a pragmatic remedy: relieving the clerical weight that adds to cognitive work while amplifying the neurologist’s capacity for insight and care.

Beyond efficiency, neurology-focused large language models herald a new integrative intelligence. As these systems evolve into large multimodal models (LMMs) capable of processing diverse data modalities, they can correlate multi-omic data (radiomic, proteomic, genomic, phenomic), fuse multimodal streams (text, imaging, waveforms), interpret digital-twin simulations, retrieve and summarize biomedical literature, and distill electronic health records into structured, comprehensible narratives [49, 51]. Increasingly, these models are coupled with retrieval-augmented generation (RAG) systems, allowing them to access curated, continuously updated corpora of guidelines, trial results, and institutional protocols rather than relying solely on static parametric memory. For each of these data streams, the model acts as both purveyor and interpreter—a never-tiring colleague who assists in recollection and reasoning within a setting of great complexity. An underappreciated capability of these emerging architectures is their capacity to explain complex machine-learning outputs and multimodal data flows. As clinical AI systems incorporate increasingly sophisticated pipelines—spanning imaging models, temporal predictors, and graph-based representations—multimodal LMMs with RAG can serve as interpreters of this complexity, translating opaque analytical steps into clinically meaningful explanations that support oversight, safety, and trust. As these capabilities mature, such systems will not replace the neurologist’s judgment; rather, they will reinforce it by clarifying what is known, suggesting what is possible, and documenting what has been done.

The promise of neurology-focused large language models is tempered by significant technical, ethical, and practical barriers [75]. The use of large language models in neurology will require continual updating of their factual foundations as well as expansion of their cognitive and procedural skill sets. Bias must be measured and mitigated, privacy protected, and trust earned through transparency and validation. Regulation must evolve to keep pace with increasingly capable systems. Multimodal data fusion—essential for integrating textual, imaging, and physiologic data—remains an unfinished scientific project [75, 76]. The alignment of structured medical knowledge with the realities of clinical practice also lags behind expectations. Ultimate success will depend on increasing technological sophistication that is coupled with sustained engagement of neurologists, data scientists, implementers, and regulators to ensure that these systems amplify, rather than erode, clinical judgment [5, 75].
